# Supplementary material for: BotCl, the First Chlorotoxin-like Peptide Inhibiting Newcastle Disease Virus: The Emergence of a New Scorpion Venom AMPs Family
Source: Molecules. 2023 May 26;28(11):4355. doi: 10.3390/molecules28114355 (PMC10254560; doi:10.3390/molecules28114355)
Supplement: Supplementary file 1 [file molecules-28-04355-s001.zip › molecules-2213379-supplementary.pdf]

molecules

MDPI

Supplementary data

Figure S1 : Hemolysis assay of venom fractions.
(A) Standard curve of water.
(B) OD mean values of crude Bot venom and BotCl

(A)

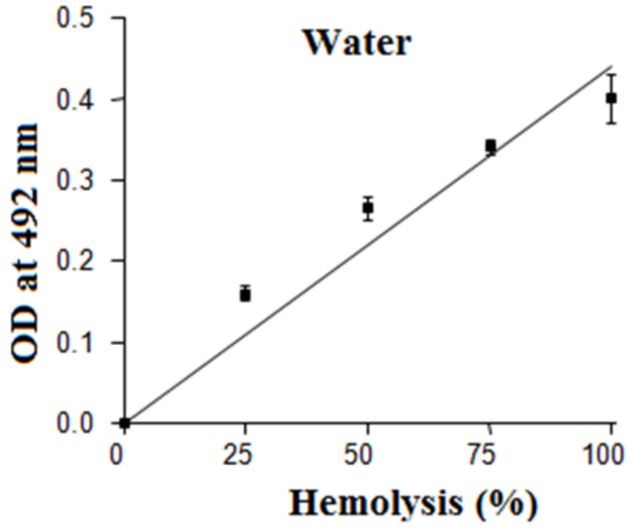

(B)

Fraction OD \ Incubation	0 h	24 h
Bot	0.0000	0.0844
BotCl	0.0000	0.0700

Table S1: binding energy and dissociation constant values of the best docking solutions

solution number	ΔG	Kd
2	-12.9	8.60E-10
3	-13.3	4.10E-10
5	-11.3	1.00E-08
6	-11.9	3.90E-09
